# Outcomes of Total Knee Arthroplasty With Simultaneous Contralateral Knee Intra-articular Corticosteroid Injection

**DOI:** 10.1016/j.artd.2026.102172

**Published:** 2026-10-01

**Authors:** Payton Jorgenson, Jennifer Rich, Drake Giese, Jonathan Webb

**Affiliations:** a Medical College of Wisconsin-Central Wisconsin, Wausau, WI, USA; b Department of Orthopaedic Surgery, Mayo Clinic Health System, Eau Claire, WI, USA; c Department of AI and Bioinformatics, Mayo Clinic Health System, Eau Claire, WI, USA; d Department of Orthopaedic Surgery, University of Nebraska Medical Center, Omaha, NE, USA

**Keywords:** Total knee arthroplasty, Intra-articular corticosteroid injection, Postoperative complication, Postoperative outcome

## Abstract

**Background:**

Corticosteroid injections are a nonoperative management option for patients with osteoarthritis (OA) of major joints. Joshy et al. and Avila et al. showed that there is an increased risk of prosthetic joint infection if corticosteroid injections are administered within 90 days of arthroplasty on the same joint. Little is known about the infection risk, postoperative complications, and pain outcomes if contralateral knees are injected perioperatively or intraoperatively

**Methods:**

This retrospective cohort study investigated patients with bilateral knee OA (n = 390) from January 2014 to December 2024 at a multilocation health system. Patients who received an intra-articular corticosteroid injection simultaneously with contrlateral total knee arthroplasty (TKA) (n = 195) were compared with controls who received no injection within 90 days of surgery (n = 195). Medical records were reviewed for comorbidities, total perioperative dexamethasone administration, total morphine milliequivalents, and postoperative complications.

**Results:**

Female patients (n = 246) were 63.1% of the total cohort. Average age of the population was 67.3 (standard deviation = 9.19). Mean body mass index was 33.2 (standard deviation = 6.39). Medical comorbidities did not significantly differ (*P* values > 0.05). Postoperative complications between groups were not statistically significant (*P* values > 0.05). Pain visual analog scale distributions were nearly identical between groups.

**Conclusions:**

Patients who underwent TKA with steroid injection the same day of the contralateral knee did not have statistically significant different outcomes compared to patients who underwent TKA without steroid injection. Thus, contralateral steroid injections at the time of TKA may be safe in patients with bilateral knee OA.

## Introduction

Osteoarthritis (OA) is a common orthopaedic disease with a large impact on healthcare [1]. Approximately 1 million hips and knees are replaced per year in the United States which is expected to increase with some projections for the rate to quadruple over the next 2 decades [2]. OA impacts patients' quality of life due to pain and decreased activity. There are nonoperative and operative approaches to managing OA. Nonoperative approaches include physical therapy, braces, analgesics, and intra-articular corticosteroid injections. Operative management is largely limited to joint arthroplasty.

Prosthetic joint infections are a postoperative complication that occurs in 1-2% of total joint arthroplasties [3]. Prosthetic joint infections have detrimental consequences with significantly increased morbidity, mortality, and increased health care costs [3]. Prior studies have established that corticosteroid injections increase the risk of periprosthetic joint infections in the perioperative period [4,5]. Studies have explored the specific contents of these intra-articular injections' impact on perioperative infections. Injections consisting of platelet-rich plasma, ketorolac, stem cell therapy, and simple aspiration are associated with an increased risk of periprosthetic joint infections [5,6].

Current orthopaedic management is to postpone joint arthroplasties of any type until 3 months have passed since the patient’s last corticosteroid injection [5,6]. However, many patients suffering from OA experience bilateral joint pain or multiple joints that cause them discomfort that would otherwise benefit from nonoperative management with intra-articular corticosteroid injections or total joint arthroplasty.

This retrospective cohort study aimed to utilize with bilateral knee OA who were undergoing total knee arthroplasties (TKAs) at a multilocation health system consisting of academic centers and community hospitals between January 2014 and December 2024.

The purpose of this study was to evaluate the postoperative complications and pain management of patients who received intra-articular corticosteroid injections at the time of contralateral TKA compared to those who did not receive intra-articular injections. We hypothesized that patients who received intra-articular corticosteroid injections at the time of contralateral TKA would have no worse postoperative complications, specifically infection risk, return to operating room, and pain management, when accounting for medical comorbidities.

## Materials and methods

Submission to the institutional review board was completed, and our study was deemed exempt. A system-wide chart review was performed using research electronic data capture (REDCap) tools [7,8]. All patients aged 30-100 years who underwent TKA (as documented by Current Procedural Terminology code 27447) from October 1, 2015 through December 31, 2024 from all healthcare system sites who were diagnosed with bilateral knee primary OA or post-traumatic OA within 1 year of the procedure were included in the review. Patients were excluded if diagnosed with inflammatory arthropathy or underwent multiple arthroplasties on the same day, such as bilateral knee arthroplasty. Of these 560 patients, 390 had complete chart records that were available for access.

The following information was obtained from the 390 patient records reviewed: comorbid medical conditions, total perioperative dexamethasone administration, corticosteroid injection medication and dosage, total morphine milliequivalents (MMEs), and postoperative complications, including return to operating room, surgical site infections, readmission rate, and pain on day of surgery and follow-ups. Dates of surgeries and follow-ups were also recorded.

A total of 195 patients who underwent TKA with same-day steroid injection of the contralateral knee were identified as the cohort group. Patients who underwent TKA without steroid injection were classified as controls. Propensity score matching, using the greedy matching method, was utilized in this observational study to reduce bias between groups by balancing covariates across groups [9]. Patients were matched one-to-one on age at surgery, body mass index (BMI), American Society of Anesthesiologists (ASA) score, and diabetes, resulting in a total of 390 patient records reviewed. Postmatching covariate balance was assessed by standardized mean differences, with an absolute difference threshold set to <0.1. Visual displays assessed outliers. Categorical variables were summarized as frequencies and percentages. Continuous variables were summarized as mean (standard deviation [SD]) or median (interquartile range). Groups were compared via the Fisher exact (categorical variables) or Kruskal-Wallis (continuous variables) tests. Multivariable logistic regression models were used to determine the strength of the association between groups, controlling for sex, age, diabetes, BMI, and ASA. Analysis was performed with SAS software, v9.4 (SAS Institute, Inc.).

## Results

Postmatching balance diagnostics showed that the largest absolute standardized mean difference was 0.094, indicating an acceptable balance of covariates between cohort and control groups. Female patients (n = 246) were 63.1% of the total cohort, with male patients (n = 144) comprising the remaining 36.9% (Table 1). The average age of the total study population was 67.3 (SD = 9.19). Mean BMI was 33.2 (SD = 6.39). The average ASA score was 2.4 (SD = 0.54). Medical comorbidities between groups were not significantly different (*P* values >0.05). Readmission within 90 days (*P* = .54), wound dehiscence (*P* = .25), surgical site infections (*P* = .69), and return to operating room (OR) (*P* = .56) rates between the injection and control groups were not statistically significant (Table 2). Pain visual analog scale (VAS) distributions assessing general pain were nearly identical across groups (Fig. 1).

**Table 1:** Demographical information of the total patient population.

Parameter	Group		Total (N = 390)	*P* value
	Cohort (N = 195)	Control (N = 195)		
				.25^a^
Female	129 (66.2)	117 (60.0)	246 (63.1)	
Male	66 (33.8)	78 (40.0)	144 (36.9)	
Age at surgery				.74^b^
Mean (SD)	67.5 (8.77)	67.1 (9.61)	67.3 (9.19)	
Median (IQR)	68.0 (62.0, 74.0)	67.0 (62.0, 74.0)	67.5 (62.0, 74.0)	
Diabetes				1.00^a^
	45 (23.1)	45 (23.1)	90 (23.1)	
BMI				.98^b^
Mean (SD)	33.1 (6.10)	33.3 (6.68)	33.2 (6.39)	
Median (IQR)	33 (29, 37)	32 (29, 38)	32 (29, 37)	
ASA score				.70^a^
Mean (SD)	2.4 (0.52)	2.4 (0.56)	2.4 (0.54)	
Median (IQR)	2 (2, 3)	2 (2, 3)	2 (2, 3)	
Medical comorbidities				
Diabetes mellitus, n (%)	23 (11.8)	23 (11.8)	46 (11.8)	1.00^a^
Coronary artery disease, n (%)	29 (14.9)	21 (10.8)	50 (12.8)	.29^a^
COPD, n (%)	6 (3.1)	9 (4.6)	15 (3.8)	.60^a^
Chronic kidney disease, n (%)	31 (15.9)	27 (13.8)	58 (14.9)	.67^a^
Other, n (%)	152 (77.9)	154 (79.0)	306 (78.5)	.90^a^

IQR, interquartile range; COPD, chronic obstructive pulmonary disease.

a Fisher exact *P* value.

b Kruskal-Wallis *P* value.

**Table 2:** Postoperative outcomes between cohort and control groups.

Outcome	Group		*P* value
	Cohort (N = 195)	Control (N = 195)	
Readmission within 90 d, n (%)			.54^a^
No	188 (96.4)	189 (97.9)	
Yes	7 (3.6)	4 (2.1)	
Missing	0	2	
Wound dehiscence within 90 d, n (%)			.25^a^
No	195 (100.0)	191 (99.0)	
Yes	0 (0.0)	2 (1.0)	
Missing	0	2	
Surgical site infections within 90 d, n (%)			.69^a^
No	190 (97.9)	191 (99.0)	
Yes	4 (2.1)	2 (1.0)	
Missing	1	2	
Return to OR for any reason (including manipulation under anesthesia), n (%)			.56^a^
No	182 (93.8)	178 (92.2)	
Yes	12 (6.2)	15 (7.8)	
Missing	1	2	
Opioids prescribed postop, n (%)			.29^a^
No	5 (2.7)	9 (4.8)	
Yes	183 (97.3)	177 (95.2)	
Missing	7	9	
Second opioid prescribed postop, n (%)			.003^a^
No	64 (34.2)	91 (49.5)	
Yes	123 (65.8)	93 (50.5)	
Missing	8	11	

a Fisher exact *P* value.

**Figure 1:**
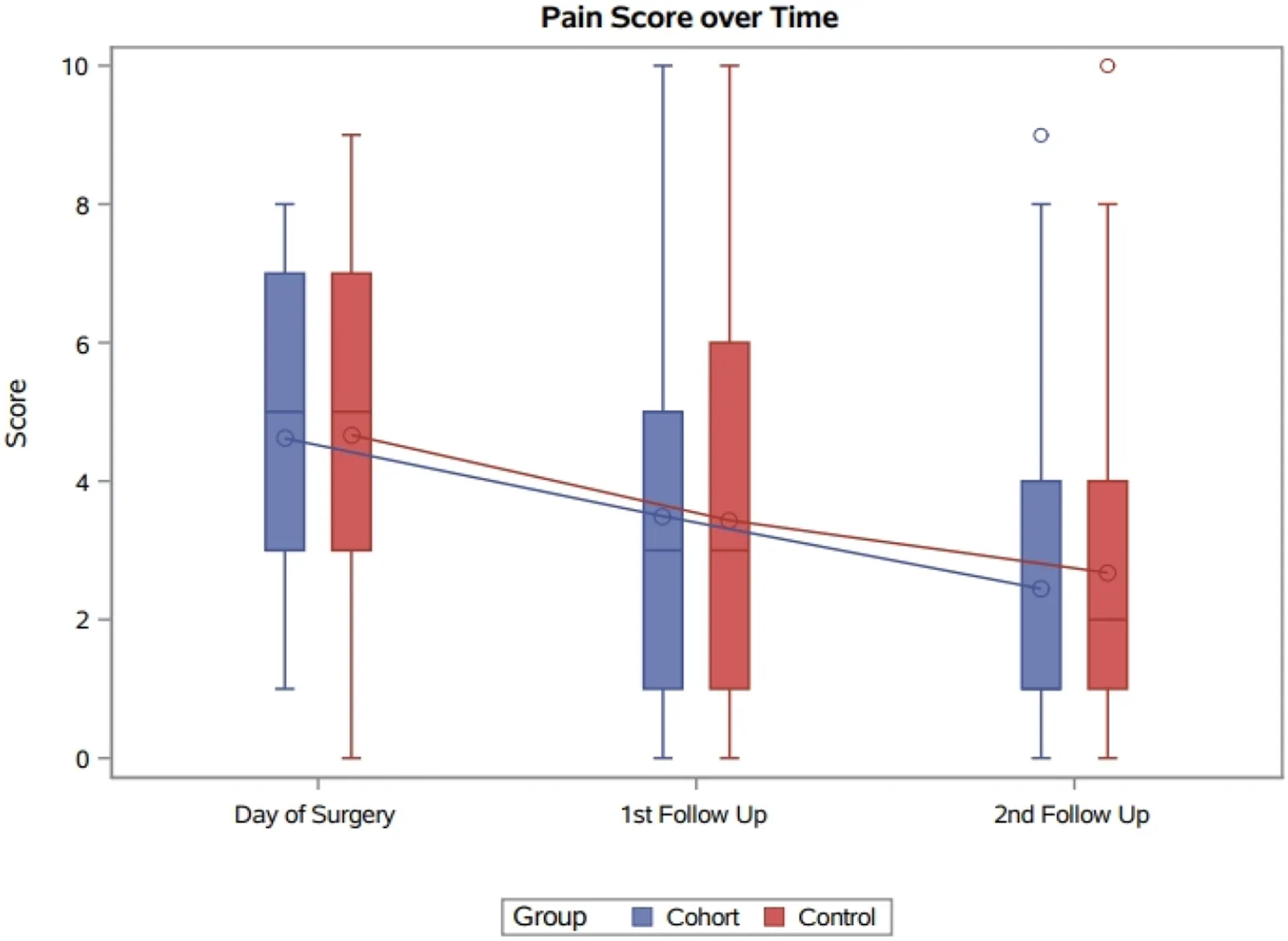
Pain score on VAS (0-10 scale) of patients who received perioperative corticosteroid injections (cohort) compared to those who did not receive perioperative corticosteroid injections (control) across initial surgery and subsequent postoperative clinic follow-ups. Group means are connected across time points.

Pain VAS scores on the day for surgery were assessed for 47 patients in the cohort group (N = 195) and 54 in the control group (N = 195). The mean (SD) was 4.6 (2.21) and 4.7 (2.46) for the cohort and control groups, respectively. At the first follow-up, mean (SD) pain VAS score for 73 patients in the cohort group (N = 195) was 3.5 (2.43), and mean (SD) pain VAS score for 60 patients in the control group (N = 195) was 3.4 (2.85). At the second follow-up, mean (SD) pain VAS score for 45 patients in the cohort group (N = 195) was 2.4 (2.06), and mean (SD) pain VAS score for 37 patients in the control group (N = 195) was 2.7 (2.42).

Patients who underwent TKA with steroid injection the same day of the contralateral knee did not have a statistically significant difference in outcomes, including infection rates or returns to the operating room compared to patients who underwent total arthroplasty without steroid injection (Table 2). Between study groups, those receiving simultaneous contralateral corticosteroid injection had significantly increased rates of receiving a second opioid prescription postoperatively (*P* < .01). Neither admission type nor perioperative administration of dexamethason were statistically significant between the two groups (Table 3).

**Table 3:** Surgical characteristics of the total patient population analyzed.

Variable	Group		*P* value
	Cohort (N = 195)	Control (N = 195)	
Admission type, n (%)			.08^a^
Same day outpatient	30 (15.6)	22 (11.4)	
Outpatient in a bed	88 (45.8)	75 (38.9)	
Inpatient	74 (38.5)	96 (49.7)	
Missing	3	2	
Perioperative administration of dexamethasone, n (%)			.11^a^
No	38 (20.7)	51 (28.3)	
Yes	146 (79.3)	129 (71.7)	
Missing	11	15	

a Fisher exact *P* value.

VAS distributions were similar across groups over time, with no clear distinction among pain scores in patients who underwent TKA with same-day steroid injection of the contralateral knee compared to patients who underwent total arthroplasty without steroid injection.

After controlling for sex, age, diabetes, BMI, and ASA, the effect of simultaneous intra-articular steroid injection to the contralateral knee at the time of TKA did not significantly increase the odds of 90-day outcomes (readmission, wound dehiscence, surgical site infections, and return to the OR) (Table 4). However, in our patient population, those receiving simultaneous intra-articular steroid injection to the contralateral knee at the time of TKA had 90% greater odds of being prescribed a second opioid postoperatively, compared to controls, accounting for sex, age, diabetes, BMI, and ASA (*P* < .01) (Table 4).

**Table 4:** Modeling the effect of simultaneous intra-articular steroid injection to the contralateral knee at the time of total knee arthroplasty and 90-day perioperative complication.

Complication	OR^a^	95% CI around OR^a^		*P* value^a^	OR^b^	95% CI around OR^b^		*P* value^b^
Readmission	1.718	0.553	5.333	.3493	1.954	0.616	1.385	0.2551
Wound dehiscence	0.187	0.019	1.875	.1541	0.133	0.013	1.875	0.0915
Surgical site infections	1.824	0.454	7.336	.3971	4.034	0.734	22.164	0.1086
Return to OR for any reason (including manipulation under anesthesia)	0.79	0.371	1.684	.5423	0.96	0.433	2.126	0.9194
Opioids prescribed postop	1.899	0.683	5.276	.2189	1.696	0.603	4.765	0.3165
Second opioid prescribed postop	1.907	1.249	2.912	.0028	1.868	1.214	2.875	0.0045

OR, odds ratio; CI, confidence interval.

a Multivariable logistic regression *P* value adjusted for sex, age at surgery, diabetes mellitus, BMI, ASA score (modeled as numeric), and perioperative administration of dexamethasone.

b Sensitivity analysis to account for the perioperative administration of dexamethasone in models.

A sensitivity analysis was conducted to assess the influence of perioperative administration of dexamethasone in our population. Results remained robust and indicate that administration of this steroid perioperatively did not have a strong influence on the outcomes in our population (Table 4). The distribution of total MME was similar between groups, with the average exposure in the cohort group being 8.3 (SD = 4.24) MME and the average exposure in the control group being 8.4 (SD = 4.36) MME.

## Discussion

There is well-described literature discussing the increased risk of infection with perioperative corticosteroid injections in the ipsilateral knee prior to total arthroplasty [5,6]. However, there is a paucity of data discussing the relationship between contralateral corticosteroid injection with simultaneous TKA.

Patients who underwent TKA with steroid injection the same day of the contralateral knee did not have statistically significantly different outcomes compared to patients who underwent total arthroplasty without steroid injection, except for patients receiving a second opioid prescription postoperatively. In our study population, there was a statistically significant difference between patients who received intraoperative corticosteroid injections receiving a second opioid prescription compared to the control population. This difference may be related to a bias by the provider in that a patient who had an injection the day of surgery may also have had significantly more pain in the contralateral knee, making an injection seem helpful. If this is true, then their postoperative pain overall may also be higher despite the injection. Further prospective studies should be performed to further elicit this difference.

As pain VAS distributions were almost identical across groups, there was no strong difference among pain scores in patients who underwent TKA with same-day steroid injection of the contralateral knee compared to patients who underwent total arthroplasty without steroid injection. However, these scales assessed general pain, not specifically pain to the operative or nonoperative knee. When comparing the VAS scores between the cohort and control group, the VAS scores are quite similar, suggesting that general pain for patients receiving same-day steroid injections theoretically do not have markedly different pain outcomes when compared to patients who do not receive same-day steroid. Further studies may be warranted to assess if there is a difference in the pain specific to the operative or nonoperative knee in patients who do and do not receive same-day steroid injection.

Limitations of the study include the recorded pain VAS that were not consistently reported for every patient, leading to limited responses for VAS scoring given the retrospective nature of the study. Another limitation with our study is the confounding administration of perioperative dexamethasone by members of the anesthesia team, which could impact the pain scoring, as well as the postoperative complications that were observed. Further studies examining the impact of perioperative dexamethasone with intra-articular corticosteroid injections may be of benefit to observe if patients do have significant differences when given intravenous dexamethasone during an orthopaedic surgical procedure. However, our study did assess the percentage of patients in each cohort that received intraoperative dexamethasone, which was not statistically significant between the control and cohort groups.

As this was a multihospital system of tertiary and community hospitals, postoperative pain programs were not completely the same. However, within the hospital system, blocks including either adductor or femoral are standard practice, as well as periarticular blocks and multimodal oral pain medications. Similarly, given that this study was retrospective and encompassed both tertiary and community hospitals, the number of surgeons who operated on the patient population is numerous. Therefore, generalizability of the data is more straightforward than just evaluation of a single center or single surgeon, as each surgeon likely had different thresholds of prescribing additional pain medications.

Another limitation of our study is the short-term follow-up. While our study did not find a difference within 90 days of follow-up, our study lacked evaluation of long-term follow-up and outcomes. Longer-term follow-up was not possible in this study; thus, future studies should include longer-term follow-up given the insidious nature of periprosthetic infection. Also, our sample size for this study is small to fully assess infection risk. The practice of simultaneous corticosteroid injections into the contralateral knee is uncommon due to surgeon fears of potential complications postoperatively. Thus, even though larger-scale numbers would be needed to fully assess infection risk, we feel that this study provides enough evidence to support the practice so that future studies can more fully assess risk and outcomes on a larger scale.

Lastly, we chose to use all available data for this retrospective review, as sample size was limited and group sizes were small. Due to the relatively rare occurrence of our primary outcome, our total sample of 390 patients likely did not give our study enough power to detect small to moderate differences between the groups. Indeed, a post hoc power analysis showed that our study only had 14.2% power to detect a difference between the 2 groups. As such, our findings should be interpreted with caution and highlight the need for additional, larger-scale studies to detect a clinically meaningful difference between patients receiving an intra-articular steroid injection to the contralateral knee at the time of TKA and patients not receiving a steroid injection at the time of surgery.

Our study found that when accounting for sex, age, diabetes, BMI, and ASA, patients receiving intra-articular steroid injection had 90% greater odds of receiving a second opioid in the postoperative period. Unfortunately, we cannot confirm if those prescriptions were filled or how much of each prescription was used. We assume that a second prescription is indicative of higher opioid use. Future studies should consider closer monitoring of opioid consumption.

## Conclusions

Intra-articular steroid injection into the contralateral knee at the time of TKA showed no significant evidence of risk of returning to the operating room or complications. Historical concerns regarding perioperative steroid use with TKA infection limited the available study population, thus impacting the overall power of the study itself. While this study does indeed have a limited power given the small sample size, it does assess the perioperative impact and short-term risk that intra-articular steroid injections in the immediate operative and perioperative time frame may have.

Prospective studies should be performed in the future to further elicit the amount of pain improvement and safety of this practice and whether this is generalizable to other injections throughout the body during the perioperative period. Our study aims to be a building block to inform future, larger studies aimed at evaluating further safety and benefits of same-day steroid injection. Given there is clinical concern with the overall short time frame of this study and the follow-up period, this study cannot rule out future infection or infection risk of this cohort. Due to the short time frame and relatively small sample size, this is an important field of orthopaedics that should be further studied. The goal of this retrospective cohort study was to evaluate and demonstrate safety in intra-articular steroid injections at the time of TKA. Future studies with larger populations and power should be considered for further exploration and evaluation of this intervention.

## CRediT authorship contribution statement

**Payton Jorgenson:** Writing – review & editing, Writing – original draft, Investigation, Data curation. **Jennifer Rich:** Writing – review & editing, Visualization, Formal analysis, Data curation. **Drake Giese:** Writing – review & editing, Writing – original draft, Investigation, Data curation. **Jonathan Webb:** Writing – review & editing, Supervision, Project administration, Methodology, Conceptualization.

## Conflicts of interest

The authors declare there are no conflicts of interest.

For full disclosure statements refer to https://doi.org/10.1016/j.artd.2026.102172.

## Appendix A Supplementary data

Conflict of Interest Statement for Giese

Conflict of Interest Statement for Jorgenson

Conflict of Interest Statement for Rich

Conflict of Interest Statement for Webb
