# Supplementary material for: Outcomes of Total Knee Arthroplasty With Simultaneous Contralateral Knee Intra-articular Corticosteroid Injection
Source: Arthroplast Today. 2026 Oct 1;41:102172. doi: 10.1016/j.artd.2026.102172 (PMC13657240; doi:10.1016/j.artd.2026.102172)
Supplement: Supplementary file 4 [file mmc3.docx]

# INDIVIDUAL CONFLICT OF INTEREST STATEMENT

***American Association of Hip and Knee Surgeons***

(Adopted from the American Academy of Orthopaedic Surgeons disclosure statement)

The following form **must be filled out completely and submitted by each author (example, 6 authors, 6 forms).**

**All items require a response. If there is no relevant disclosure for a given item, enter "*None*.”**

**Manuscript Title: Outcomes of Total Knee Arthroplasty with Simultaneous Contralateral Knee Intraarticular Corticosteroid Injection**

1.	Royalties from a company or supplier (The following conflicts were disclosed)

*None*

2.	Speakers bureau/paid presentations for a company or supplier (The following conflicts were disclosed)

*None*

3A.	Paid employee for a company or supplier (The following conflicts were disclosed)

*None*

3B.	Paid consultant for a company or supplier (The following conflicts were disclosed)

*None*

3C.	Unpaid consultants for a company or supplier (The following conflicts were disclosed)

*None*

4.	Stock or stock options in a company or supplier (The following conflicts were disclosed)

*None*

5.	Research support from a company or supplier as a Principal Investigator (The following conflicts were disclosed)

*None*

6.	Other financial or material support from a company or supplier (The following conflicts were disclosed)

*None*

7.	Royalties, financial or material support from publishers (The following conflicts were disclosed)

*None*

8.	Medical/Orthopaedic publications editorial/governing board (The following conflicts were disclosed)

*None*

9.	Board member/committee appointments for a society (The following conflicts were disclosed)

*None*

**Each author must sign AND print or type his/her name, date and submit a separate form**

In addition, one BLINDED Conflict of Interest form (no author names used) should be submitted per manuscript with all author disclosures.

Jennifer R Rich	1/23/2026

Author Name (Print or Type)	Author Signature	Date
